# Novel nitrogen doped graphene sponge with ultrahigh capacitive deionization performance

**DOI:** 10.1038/srep11225

**Published:** 2015-06-11

**Authors:** Xingtao Xu, Zhuo Sun, Daniel H. C. Chua, Likun Pan

**Affiliations:** 1 Engineering Research Center for Nanophotonics & Advanced Instrument, Ministry of Education, Shanghai Key Laboratory of Magnetic Resonance, Department of Physics, East China Normal University, Shanghai 200062, China; 2 Department of Materials Science and Engineering, National University of Singapore, Singapore 117574

## Abstract

As water shortage has become a serious global problem, capacitive deionization (CDI) with high energy efficiency and low cost, is considered as a promising desalination technique to solve this problem. To date, CDI electrodes are mainly made up of porous carbon materials. However, the electrosorption performance obtained by now still cannot meet the demand of practical application. Therefore, a rationally designed structure of electrode materials has been an urgent need for CDI application. Here, a novel nitrogen-doped graphene sponge (NGS), with high specific surface area and rationally designed structure was fabricated, and used as CDI electrodes for the first time. The results show that NGS exhibits an ultrahigh electrosorption capacity of 21.0 mg g^−1^ in ∼500 mg L^−1^ NaCl solution, and to our knowledge, it is the highest value reported for carbon electrodes in similar experimental conditions by now. NGS in this work is expected to be a promising candidate as CDI electrode material.

As the shortage of freshwater has become one of the most serious threats to humanity, it is generally recognized that desalination of saline water is the most effective solution to increase the supply of freshwater^1^. Currently, desalination technologies, such as reverse osmosis, electrodialysis and evaporation, have been widely used to supply freshwater. However, huge energy consumption and high costs have limited their large scale applications in the developing world where energy and capital supplies are very limited^2^,^3^. Therefore, seeking an innovative desalination technology with high energy efficiency, low cost, easy regeneration and non-secondary pollution has been urgent for current society. Recently, capacitive deionization (CDI) is considered as one of the most promising techniques that meet all of the above requirements^4^,^5^,^6^,^7^, because it can be conducted at ambient conditions and low voltages (<2 V) without secondary waste, and doesn’t require high-pressure pumps, membranes, distillation columns, or thermal heaters. As an electrochemical water treatment method, CDI is developed based on the principle of electric double-layer (EDL) capacitor. With an external electrostatic field supply between two electrodes, the charged ions can move toward oppositely charged electrodes, and be attracted within the EDL formed between the solution and electrode interface (Supplementary Figure 1). On the basis of this mechanism, the ion adsorption capacity of an electrode is directly related to the physical properties and internal structure of the electrode materials, such as surface area, electrical conductivity and pore size.

Generally, porous carbon materials with high surface area and good electrical conductivity, such as activated carbon (AC)^8^,^9^,^10^, carbon aerogel (CA)^11^,^12^, carbon nanofibre (CNF)^13^,^14^, carbon nanotube (CNT)^15^,^16^ and mesoporous carbon (MC)^17^,^18^, have been widely used as the CDI electrodes. Besides these carbon species, graphene with a flexible planar structure (ultrathin layer), high specific surface area (theoretically ~2600 m^2^ g^−1^) and superior electron mobility (theoretically ~2.5 × 10^5^ cm^2^ V^−1^ s^−1^ at room temperature), has been theoretically and experimentally demonstrated to possess good CDI performance. However, due to the strong agglomeration between graphene nanosheets during the reduction process, pristine graphene (PG) has relatively low specific surface area, and thus shows low electrosorption capacity of 0.45–1.85 mg g^−1^,^19^,^20^,^21^ which hampers its application in CDI. To date, two strategies have been applied to prevent the agglomeration between graphene nanosheets, and thus enhance their CDI performance. On the one hand, by introducing some guest materials, such as pyridine^22^, CNT^23^,^24^, MC^18^, AC^25^ and resol^26^,^27^ as “spacers” between graphene sheets to form a sandwich structure, the electrosorption capacity is enhanced obviously (0.83–3.23 mg g^−1^) compared with PG. Nevertheless, it is difficult to keep the “spacers” dispersed uniformly between the graphene sheets, so the aggregation of graphene still partly exists in these composites^28^. On the other hand, efforts are made to design and optimize the structure of graphene for its practical application in CDI. Towards this aim, three-dimensional (3D) graphene structures including 3D macroporous graphene architectures^29^, 3D graphene-based hierarchically porous carbon composites^30^, sponge-templated prepared graphene^31^, 3D graphene/metal oxide hybrids^32^ and our recently reported graphene sponge (GS)^33^ have been fabricated and used as CDI electrodes. There is no denying the fact that these tailored 3D graphene structures can prevent the agglomeration of graphene sheets, enlarge the specific surface area, and thus improve the electrosorption capacity up to 3.9–15.1 mg g^−1^. However, the synthetic strategies of some of these materials are relatively complicated, time-consuming, and practically high-cost. Moreover, further enhancement of electrosorption capacity is still necessary in order to meet the practical demand in desalination application. Hence, essential efforts are still needed to seek a facile and low-cost strategy to fabricate novel graphene structure with high electrosorption capacity for practical applications of CDI.

In this work, we present a novel, facile and low-cost strategy for large-scale production of nitrogen doped graphene sponge (NGS). More importantly, when used as CDI electrodes, the obtained NGS exhibits an ultrahigh electrosorption capacity of 21.0 mg g^−1^ in NaCl solution with an initial concentration of ∼500 mg L^−1^, which is much higher than those of other carbon based electrodes in similar experimental conditions reported in the literatures by now.

## Results

### Fabrication strategy and morphology of NGS

**Figure 1:**
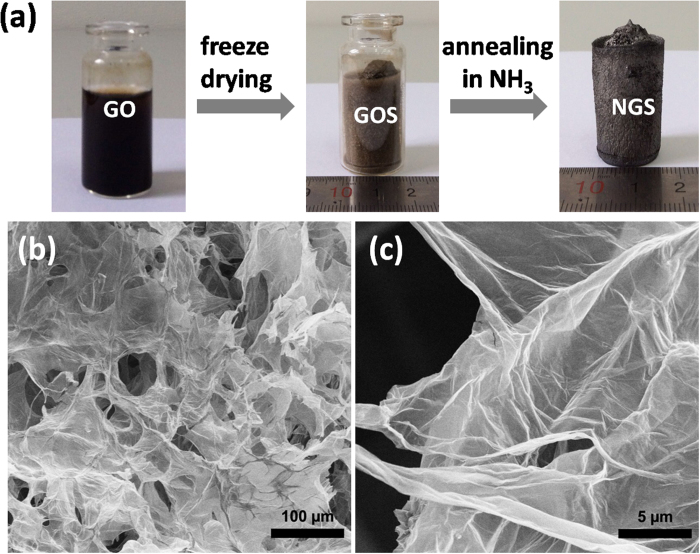
(**a**) Schematic illustration of the procedure for the preparation of NGS. (**b**,**c**) SEM images showing the interconnected, porous 3D framework structure of NGS. (**b**) interconnecting porous network between the graphene layers, scale bar: 100 μm; (**c**) corrugated and scrolled graphene nanosheets, scale bar: 5 μm.

Figure 1a illustrates the method for the fabrication of NGS. Graphene oxide sponge (GOS) was produced by freeze-drying process in which the strong interaction of graphene oxide (GO) in water plays an important role. In this process, the interaction of GO was strong enough so that GO solution could be directly frozen to produce the sponge. After annealing in NH_3_, NGS was obtained. Compared with the methods reported for the fabrication of 3D graphene architectures^29^,^30^,^31^,^32^, the strategy in this work is more simple, low-cost and easy for large-scale production. The morphology of NGS was investigated by scanning electron microscopy (SEM), as presented in Figure 1b and c. As shown, NGS exhibits an interconnected porous 3D framework of randomly oriented crinkly sheets. The high magnification SEM image in Figure 1c reveals clearly that the obtained 3D NGS structure is comprised of corrugated and scrolled graphene nanosheets. These curls and wrinkles clearly act to prevent graphene sheets from restacking together with each other. Furthermore, the morphologies of GS and PG were also investigated by SEM (Supplementary Figure 2). It can be seen that the morphology of GS is similar to that of NGS (Supplementary Figure 2a and b), whereas PG displays a plane structure with some graphene sheets stacking to graphite platelets due to the strong Van der Waals forces among individual graphene nanosheets (Supplementary Figure 2c and d). It is known that the EDL on the large exposed area can provide sufficient accessible sites for ion accumulation and the space between the pores can serve as an efficient channel for fast mass transfer^31^. Therefore, NGS with interconnected porous structures is expected to have high specific surface area and large pore space, which are desirable for high performance CDI.

### Characterization of NGS

**Figure 2:**
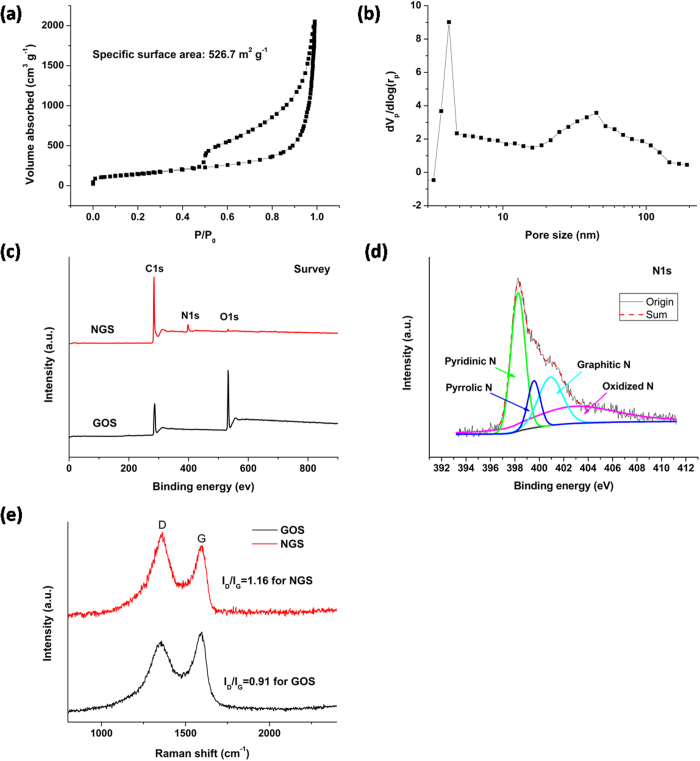
Characterization of NGS. Nitrogen sorption isotherm (**a**) and pore size distribution (**b**) of NGS; (**c**) XPS spectra of GOS and NGS; (**d**) high-resolution N1s XPS of NGS; (**e**) Raman spectra of GOS and NGS.

The structure and composition of NGS were characterized and compared with GS and PG (Figure 2 and Supplementary Figure 3). It is known that large specific surface area and suitable pore structure is crucial for electrode materials. Therefore, nitrogen adsorption–desorption analysis was performed, and the results were shown in Figure 2a and b. NGS shows a typical type IV hysteresis loop as defined by IUPAC, which is characteristic of mesoporous materials^34^. The hysteresis loop which appears at low relative pressure (0.4–0.8) indicates the presence of mesopores and at high relative pressure (0.8–1.0) is attributed to macropores^29^. As calculated, NGS displays a high specific surface area of 526.7 m^2^ g^−1^ with a pore volume of 3.13 cm^3^ g^−1^, much higher than those of GS (356.0 m^2^ g^−1^ and 1.51 cm^3^ g^−1^), PG (150.5 m^2^ g^−1^ and 0.83 cm^3^ g^−1^) and other graphene structures in the literatures^19^,^21^,^29^,^30^,^31^,^32^. Moreover, the pore size distribution of NGS (Figure 2b) determined by the Barret-Joyner-Halenda method was compared with those of GS and PG (Supplementary Figure 3c and d). Obviously, NGS shows much more mesopores and macropores than GS and PG. The pore size distribution of NGS is in the range of 3.5–100 nm with double-peak pore diameters of 4.5 and 45 nm. As known, the interconnected macropores within graphene framework are favorable for buffering ions to shorten the diffusion distance from the external electrolyte to the interior surface^29^,^35^, and the mesopores in thin walls can enhance the ion transport and the electrosorption capacity^35^,^36^. Therefore, NGS is expected to be a promising candidate as CDI electrode materials.

To measure the nitrogen content and examine chemical change caused by the nitrogen doping, X-ray photoelectron spectroscopic (XPS) measurements were carried out. As expected, the XPS survey spectrum (Figure 2c) for the GOS shows only C1s and O1s peaks at about 285 and 534 eV, respectively. The high content of O element (34.7 at.%, Figure 2c) suggests that the oxygen-rich groups exist within the GOS. Upon nitrogen doping, a N1s peak appears at about 398 eV (8.5 at.% N, Figure 2c), accompanied by a significant decrease in the oxygen content (0.76 at.%, Figure 2c). The change of nitrogen and oxygen contents before and after nitrogen doping indicates the reduction of GO induced by the doping with nitrogen^37^. Moreover, the oxygen contents in GS (7.7 at.%, Supplementary Figure 3e) and PG (8.4 at.%, Supplementary Figure 3e) are also lower than that in GOS (34.7 at.%, Figure 2c), indicating the efficient reduction of GO to graphene by annealing. The lower oxygen content in NGS than in GS and PG demonstrates that nitrogen doping increases the reduction efficiency of GO^37^. The high resolution N1s spectrum of NGS shows four peaks at 398.2, 399.5, 400.9 and 402.8 eV (Figure 2d), corresponding to pyridinic nitrogen, pyrrolic nitrogen, graphitic nitrogen and oxidized nitrogen, respectively. The XPS analysis indicates that nitrogen atoms have successfully doped in graphene backbone. Previous work has reported that the electrical conductivity of graphene can be improved through nitrogen doping^38^. Therefore, the NGS is expected to have a high electrical conductivity, which is beneficial to the electron transfer during the electrosorption process. Figure 2e shows the Raman spectra of GOS before and after the nitrogen doping. The D-band of graphitic materials is a measure of disorder and arises due to the breathing mode of k-point phonons of A_1g_ symmetry while the G-band is associated with conjugated structure of sp^2^ carbon domains. As shown in Figure 2e, the NGS shows a much higher ratio (1.16) of the peak intensities of the D and G band (I_D_/I_G_) than GOS (0.91) because of the structural distortion induced by the nitrogen doping^39^ and the loss of carbon atoms by the decomposition of oxygen-containing groups^40^. Moreover, the I_D_/I_G_ is used to estimate the defect lattices and degree of disorders in graphene. Clearly, the I_D_/I_G_ of NGS is higher than those of GS (1.05) and PG (0.93), as shown in Supplementary Figure 3c. As well known, the presence of defects can generate more accessible surface area and cause an increase in ability for the accumulation of charges, which is beneficial to the charge transfer in the adsorption process^41^,^42^.

### High specific capacitance and low charge transfer resistance

**Figure 3:**
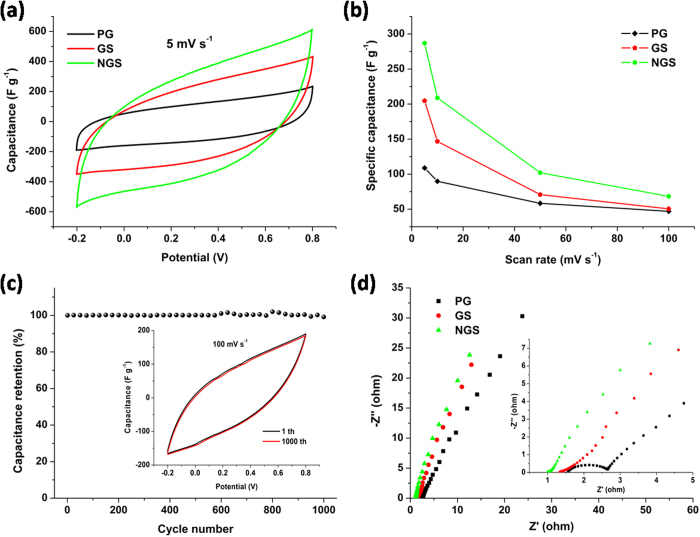
Electrochemical properties of NGS in 1 M NaCl solution. (**a**) CV curves of PG, GS and NGS at a scan rate of 5 mV s^−1^; (**b**) the specific capacitances of PG, GS and NGS at different scan rates; (**c**) cycling performance of NGS at a scan rate of 100 mV s^−1^. Inset presents the CV curves before and after 1000 cycles. (**d**) Nyquist plots of NGS, GS and PG electrodes. Inset is the corresponding expanded high-frequency region of the plots.

The electrochemical performance of NGS was investigated and compared with GS and PG. Figure 3a shows the cyclic voltammetry (CV) curves of NGS, GS and PG at a scan rate of 5 mV s^−1^ in 1 M NaCl solution with a potential range from −0.2 to 0.8 V. Apparently, the CV curves of all these materials exhibit a typical capacitor behaviour with a nearly rectangular shape, which suggests that the CV behavior results from EDL due to the Coulombic interactions, rather than the electrochemical reduction/oxidation reactions^29^. The specific capacitance of NGS electrode calculated from the CV curve is 286.86 F g^−1^, which is much higher than those of GS (204.66 F g^−1^) and PG (108.83 F g^−1^). Figure 3b further reveals that, at any scan rates ranging from 5 mV s^−1^ to 100 mV s^−1^, NGS possesses substantially higher specific capacitances than those of GS and PG. The higher specific capacitance of NGS electrode can be ascribed to its higher specific surface area and pore volume, fewer layered and higher disordered graphitic structure, surperb electrical conductivity as well as possible pseudocapacitance contribution from nitrogen doping^43^. Furthermore, the superior electrochemical property of NGS is also highlighted by its excellent cyclic stability. As shown in Figure 3c, there is no change in the specific capacitance of NGS after 1000 cycles, and its CV curve keeps the original shape. These results indicate that our NGS is expected to become not only a superb CDI electrode material, but also a promising candidate as supercapacitor electrode materials.

Electrochemical impedance spectroscopy (EIS) analysis has been recognized as one of the principal methods to examine the electrical conductivity of a carbon electrode. The Nyquist profiles of NGS, GS and PG electrodes in 1 M NaCl aqueous solution are presented in Figure 3d. It can be obviously seen that the plots display similar shapes, consisting of a linear trait at the low frequency region and a small quasi-semicircle at the high frequency one. The small quasi-semicircle at the high-frequency region is derived from the double layer capacitance (C_dl_) in parallel with the charge transfer resistance (R_ct_)^23^. The R_ct_ can be obtained from the diameter of the semicircle. The R_ct_ for NGS is around 0.09 Ω that is much lower than those of GS (0.27 Ω) and PG (1.12 Ω), showing that the 3D porous structure of NGS can facilitate the charge transfer.

### Ultrahigh CDI performance

**Figure 4:**
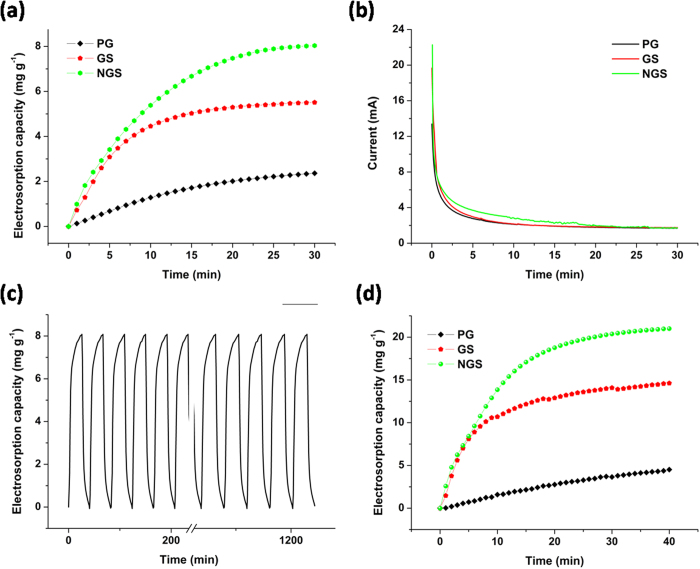
Electrosorption performance of NGS in NaCl solution. (**a**) Electrosorption capacity and (**b**) current transient for NGS, GS and PG electrodes over 30 minutes in NaCl solution with an initial concentration of ∼50 mg L^−1^ at an applied voltage of 1.5 V; (**c**) electrosorption and regeneration cycles for NGS in NaCl solution with an initial concentration of ∼50 mg L^−1^ at an applied voltage of 1.5 V; (**d**) electrosorption capacity of NGS, GS and PG electrodes over 40 minutes in NaCl solution with an initial concentration of ∼500 mg L^−1^ at an applied voltage of 1.2 V.

To determine the electrosorption performance of NGS, GS and PG electrodes, batch mode CDI experiments were carried out in NaCl solution with an initial concentration of ∼50 mg L^−1^ at an applied potential of 1.5 V. The current variations were recorded simultaneously and independently at each experiment. Figure 4a,b show the electrosorption performances and typical current responses of NGS, GS and PG, respectively. Once the electric field was applied, the adsorption amount increased sharply. It can be noted that NGS shows an electrosorption capacity of 8.04 mg g^−1^, which is much higher than those of GS (5.51 mg g^−1^) and PG (2.36 mg g^−1^). To our knowledge, it is highest value reported for graphene structures as CDI electrodes by now (0.88–4.95 mg g^−1^)^19^,^21^,^22^,^29^,^31^,^34^,^44^. Furthermore, charge efficiency (*Λ*) is used to study the electrosorption behaviors of these electrode materials, and the values of NGS, GS and PG are 0.60, 0.49 and 0.27, respectively. Obviously, NGS shows higher charge efficiency than GS and PG. Besides the electrosorption capacity and charge efficiency, the reversibility is another important factor for the actual application of CDI electrode materials. As shown in Figure 4c, NGS electrode can be completely regenerated and reused for over 30 cycles without any declination.

In the actual case, if the Total Dissolved Solids (TDS, measured in mg L^−1^) is higher than 500 mg L^−1^, water is not suitable for drinking. The electrosorption experiments were further performed in NaCl solution with an initial concentration of ∼500 mg L^−1^. The electrosorption capacity of NGS is calculated to be 21.0 mg g^−1^, which is about 1.4 and 4.6 times of the ones of GS (14.6 mg g^−1^) and PG (4.5 mg g^−1^), respectively. Notably, the electrosorption capacity of NGS also outperforms other CDI electrode materials in similar experimental conditions reported in the literatures (Supplementary Table 1).

## Discussion

The ultrahigh electrosorption capacity of NGS is mainly due to the following reasons: (i) the 3D porous structure of NGS with an enhanced specific surface area (526.7 m^2^ g^−1^) and an increased pore volume (3.13 cm^3^ g^−1^) provides short ion diffusion pathways and more available spaces to accommodate ions during the electrosorption; (ii) the fewer layered and higher disordered graphitic structure of NGS facilitates the charge transfer; (iii) nitrogen doping increases the pseudo-capacitance^43^, improves the electrical conductivity of graphene^38^ and the wettability of the interface between the electrolyte and the electrodes^45^, which can decrease the inner resistance of electrodes and help to improve the electrosorption performance of NGS. Consequently, NGS should be a promising candidate as CDI electrode material for practical applications.

In summary, NGS was prepared by directly freeze-drying GO followed by annealing in NH_3_ atmosphere, rather than using template method or hydrothermal method described in previous reports. Due to the simplicity of assembly process in our strategy and the general availability of GO, large-scale production of NGS with desired shapes can be readily accessible. Furthermore, NGS was applied as CDI electrode for the first time, and shows a ultrahigh electrosorption capacity of 21.0 mg g^−1^ when the initial NaCl concentration is ∼500 mg L^−1^, which is about 1.4 and 4.6 times of those of GS (14.6 mg g^−1^) and PG (4.5 mg g^−1^). Notably, this value is also higher than those of other electrodes reported in the literatures by now. The enhanced electrosorption performance of NGS is ascribed to its large accessible surface area and pore volume, low charge transfer resistance, superior pore structure and increased specific capacitance by nitrogen doping. It is expected that this work will not only provide a promising candidate as CDI electrode material for practical applications, but also offer the potential to develop high performance material applied in other energy storage areas including supercapacitors and batteries.

## Methods

### Preparation of GO

GO was prepared according to the method reported in our previous work^19^. In brief, graphite powder was put into a solution of concentrated nitric acid and sulphuric acid (1: 2 in volume) and kept at 80 °C for 5 h. The mixture was cooled to room temperature, diluted with deionized (DI) water and left overnight. Then, the reaction vessel was immersed in an ice bath, and potassium permanganate was added slowly. Successively, the mixture was stirred and left for 2 h. Then, after the dilution with DI water, 30% H_2_O_2_ was added into the mixture, and the color of mixture changed into brilliant yellow along with bubbling. Finally, the mixture was filtered and washed with HCl aqueous solution (1: 10 in volume), DI water and ethanol, respectively. Finally, the obtained GO was dried in vacuum oven at 60 °C for 24 h.

### Preparation of NGS

In a typical process, GO solution (~4 mg mL^−1^) in a vial was frozen by placing it in a freezer at a freezing temperature of −18 °C for 2 days. After the GO solution was completely frozen, the vial was moved to a freeze-dryer and dried at a sublimating temperature of −53 °C and a pressure <10 pa for 3 days to get the GOS. Finally, NGS was obtained by annealing GOS in a tubular furnace at 800 °C under NH_3_ flow for 3 h. For comparison, GS was fabricated by placing GOS in a tubular furnace at 800 °C under nitrogen flow for 3 h. PG was fabricated by placing GO in a tubular furnace at 800 °C under nitrogen flow for 3 h.

### Characterization

The surface morphology and structure of the samples were characterized by SEM (JEOL JSM-LV5610). The pore size distribution and Brunauer–Emmett–Teller specific surface area were deduced from the nitrogen physical adsorption measurement data which were obtained using ASAP 2010 Accelerated Surface Area and Porosimetry System (Micrometitics, Norcross, GA). Raman spectra were obtained by Renishaw inVia microscope. A He-Ne laser (633 nm) was used as the light source for excitation. XPS measurement was performed on an Imaging Photoelectron Spectrometer (Axis Ultra, Kratos Analytical Ltd.) with a monochromatic Al Ka X-ray source. CV and EIS measurements were carried out in 1 M NaCl solution by using Autolab PGSTAT 302N electrochemical workstation in a three-electrode mode, including a standard calomel electrode as reference electrode and a platinum foil as counter electrode. The specific capacitance (*C*, F g^−1^) can be obtained from CV curves using the following equation:
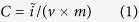
where 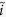 is the average current (A), *v* is the scan rate (V s^−1^) and *m* is the mass of electrodes (g).

### Electrosorption experiments

The electrodes were prepared by mixing 80 wt% of samples, 10 wt% of acetylene black, and 10 wt% of polyvinyl alcohol slurry. The mixtures were pressed onto graphite papers and dried in vacuum oven at 60 °C overnight.

The CDI experiments were investigated by batch-mode electrosorption experiments with a continuously recycling system, as described in our previous work^46^. In each experiment, the analytical pure NaCl solution with a volume of 50 mL was employed as the target solution and the flow rate was 27 mL min^−1^. A direct voltage of 1.5 V was applied on the opposite electrodes. The initial concentration of NaCl solution was ∼50 mg L^−1^, and the solution temperature was kept at 298 K. The relationship between conductivity and concentration was obtained according to a calibration table made prior to the experiment^19^. The concentration variation was continuously monitored and measured at the outlet of the unit cell by using an ion conductivity meter.

In our experiment, the electrosorption capacity (*Γ*, mg g^−1^) was defined as follows:
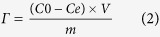
where *C*_0_ and *C*_e_ are initial and final NaCl concentrations (mg L^−1^), *V* is the volume of NaCl solution (L) and *m* is the total mass of the electrodes (g).

Charge efficiency (*Λ*) is a functional tool to gain insight into the double layer formed at the interface between the electrode and the solution^7^,^47^,^48^,^49^,^50^, as calculated according to the following equation:
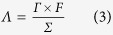
where *F* is the Faraday constant (96485 C mol^−1^), *Γ* is the electrosorption capacity (mol g^−1^) and *Σ* (charge, C g^−1^) is obtained by integrating the corresponding current.

## Additional Information

**How to cite this article**: Xu, X. *et al.* Novel nitrogen doped graphene sponge with ultrahigh capacitive deionization performance. *Sci. Rep.* **5**, 11225; doi: 10.1038/srep11225 (2015).

## Supplementary Material

Supplementary Information
