# Supplementary material for: Novel nitrogen doped graphene sponge with ultrahigh capacitive deionization performance
Source: Sci Rep. 2015 Jun 11;5:11225. doi: 10.1038/srep11225 (PMC4463025; doi:10.1038/srep11225)
Supplement: Supplementary Information [file srep11225-s1.doc]

Novel nitrogen doped graphene sponge with ultrahigh capacitive deionization performance

Xingtao Xu^a^, Zhuo Sun^a^, Daniel H. C. Chua^b^ and Likun Pana[[1]](#footnote-2)*

^a^Engineering Research Center for Nanophotonics & Advanced Instrument, Ministry of Education, Shanghai Key Laboratory of Magnetic Resonance, Department of Physics, East China Normal University, Shanghai 200062, China

^b^Department of Materials Science and Engineering, National University of Singapore, Singapore 117574

**Supplementary Figures**

**
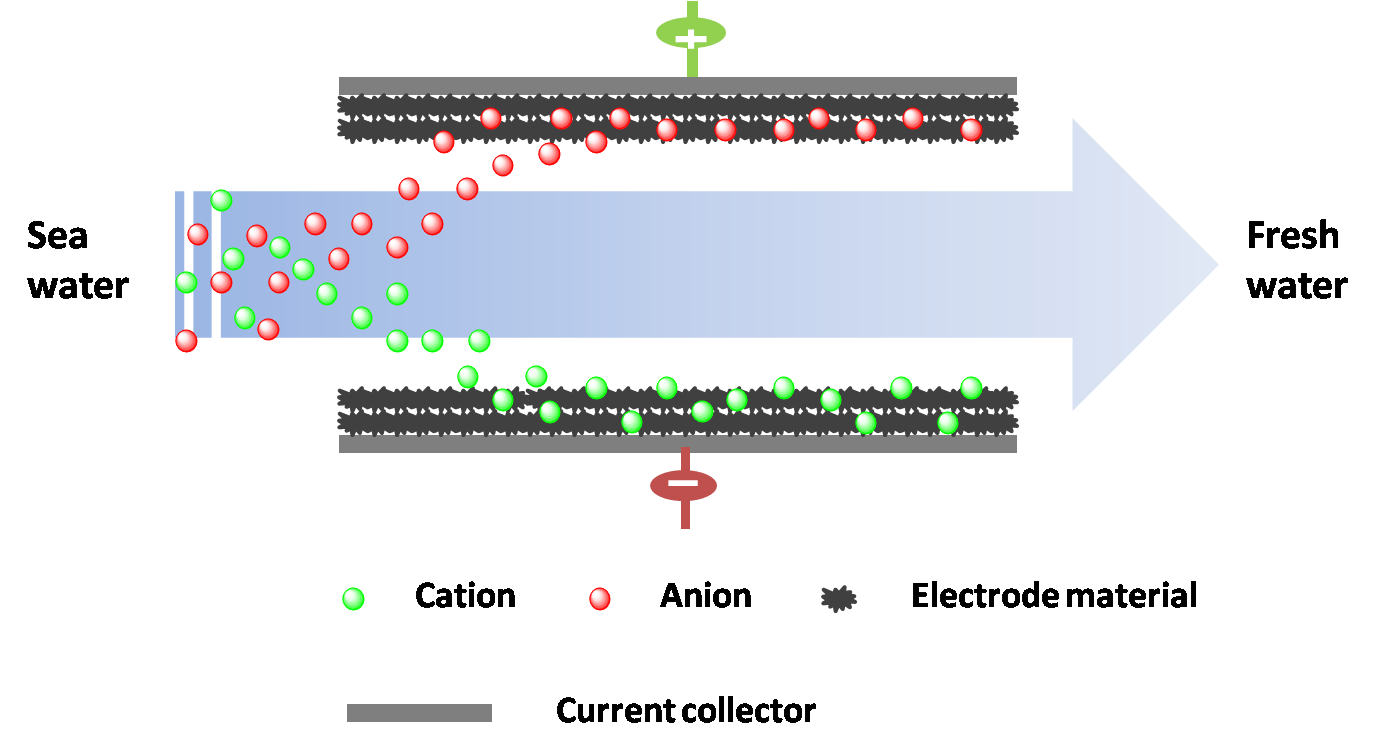
**

**Supplementary Figure 1.** Schematic diagram of the CDI process.


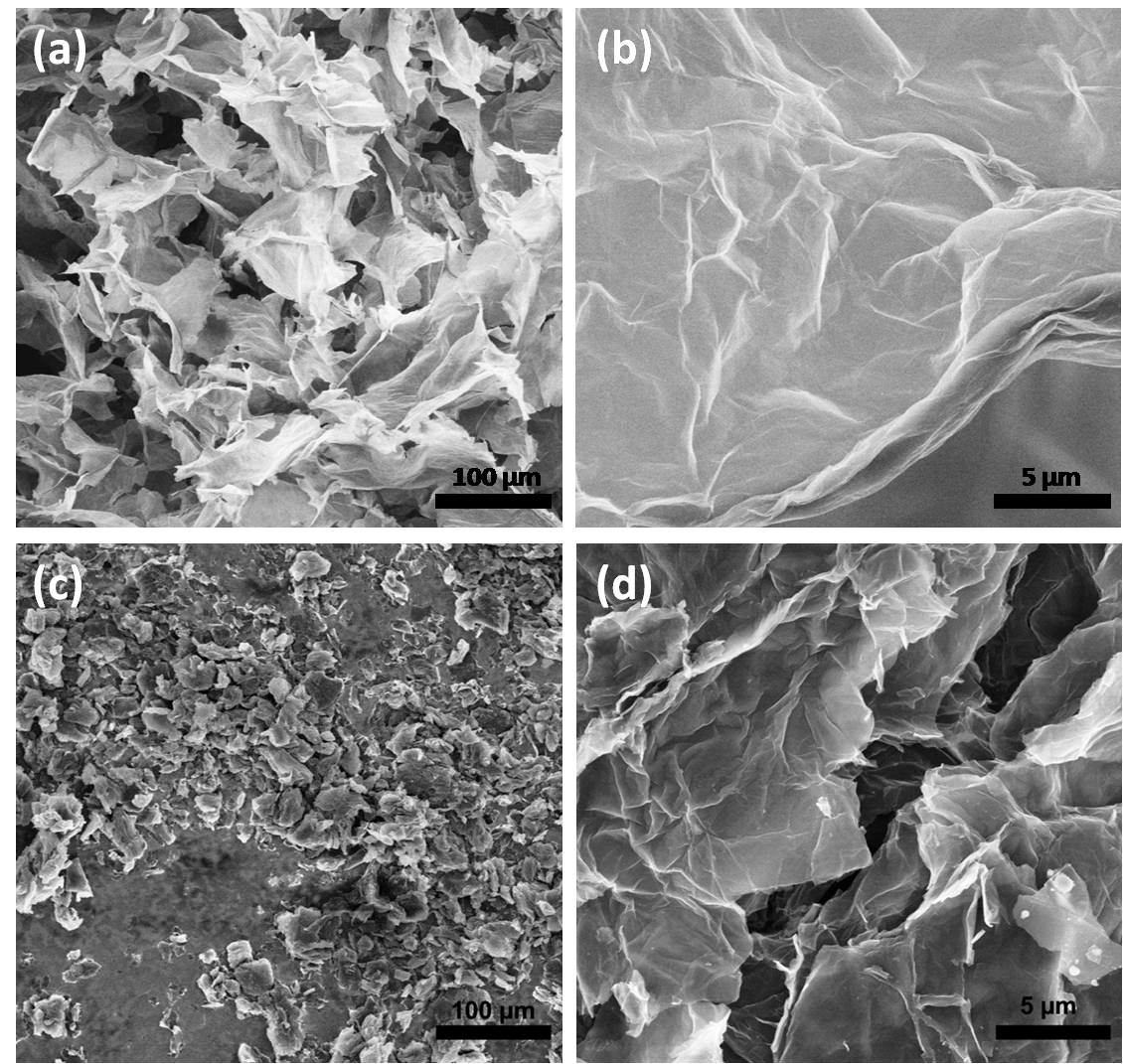


**Supplementary Figure 2.** (a-d) SEM images of GS (a, b) and PG (c, d) at different magniﬁcations. Scale bars: (a, c) 100 μm, (b, d) 5 μm.


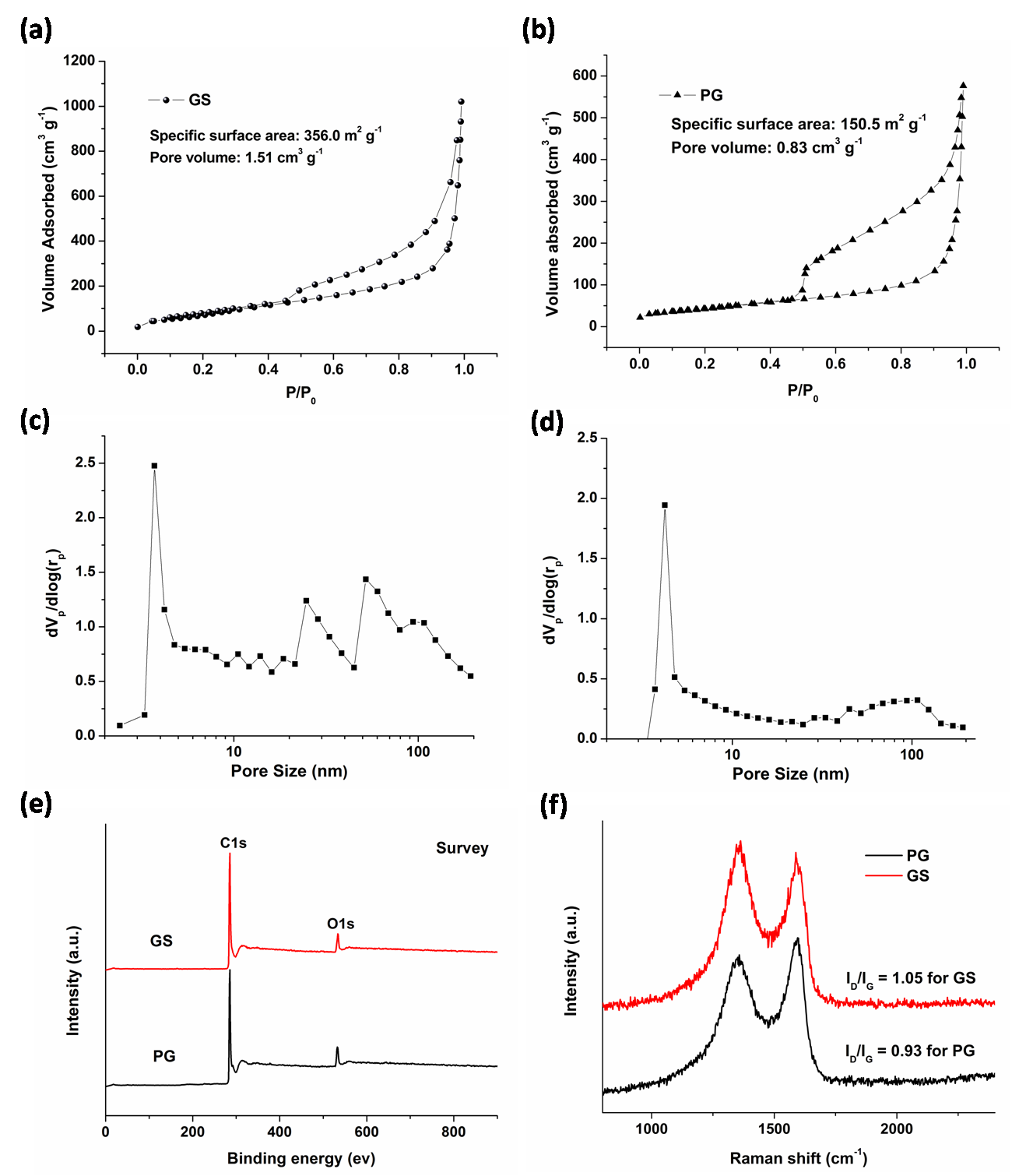


**Supplementary Figure 3.** (a-d) Nitrogen sorption isotherm and pore size distribution of (a, c) GS and (b, d) PG;[^1^](#_ENREF_1) (e) XPS spectra of GS and PG; (f) Raman spectra of GS and PG.

**Supplementary Tables**

**Supplementary Table 1.** Comparison of electrosorption capacities of various carbon electrodes.[^1^](#_ENREF_1)

| Sample | Applied voltage (V) | Initial NaCl concentration (mg L^-1^) | Electrosorption capacity (mg g^-1^) | Specific surface area (m^2^ g^-1^) |
| --- | --- | --- | --- | --- |
| AC[^2^](#_ENREF_2) | 1.2 | ~500 | 9.72 | 1153 |
| Multi-walled CNT[^3^](#_ENREF_3) | 1.2 | ~3000 | 1.7 | 129.4 |
| MC[^4^](#_ENREF_4) | 1.2 | ~4460 | 14.5 | 488 |
| Microporous CA monoliths[^5^](#_ENREF_5) | 1.25 | ~2900 | 9.6 | ~500 |
| CA[^6^](#_ENREF_6) | 1.3 | ~2000 | 7.1 | 113 |
| CNT/graphene composite[^7^](#_ENREF_7) | 1.2 | ~500 | 1.4 | 438.6 |
| AC/graphene composite[^8^](#_ENREF_8) | 1.2 | ~500 | 2.94 | 779 |
| CNT[^9^](#_ENREF_9) | 1.2 | ~3500 | 9.35 | 153 |
| Graphene aerogel[^10^](#_ENREF_10) | 1.2 | ~500 | 9.9 | - |
| Graphene/TiO_2_ aerogel hybrid[^10^](#_ENREF_10) | 1.2 | ~500 | 15.1 | 187.60 |
| NGS (this work) | 1.2 | ~500 | 21.0 | 526.7 |
| GS (this work) | 1.2 | ~500 | 14.6 | 356.0 |
| PG (this work) | 1.2 | ~500 | 4.5 | 150.5 |

**References**

1 Xu, X.-T. *et al.* Facile synthesis of novel graphene sponge for high performance capacitive deionization. *Sci. Rep.* **5**, 8458 (2015).

2 Chen, Z., Song, C., Sun, X., Guo, H. & Zhu, G. Kinetic and isotherm studies on the electrosorption of NaCl from aqueous solutions by activated carbon electrodes. *Desalination* **267**, 239-243 (2011).

3 Dai, K., Shi, L., Fang, J., Zhang, D. & Yu, B. NaCl adsorption in multi-walled carbon nanotubes. *Mater. Lett.* **59**, 1989-1992 (2005).

4 Tsouris, C. *et al.* Mesoporous carbon for capacitive deionization of saline water. *Environ. Sci. Technol.* **45**, 10243-10249 (2011).

5 Suss, M. E. *et al.* Capacitive desalination with flow-through electrodes. *Energy Environ. Sci.* **5**, 9511-9519 (2012).

6 Xu, P., Drewes, J. E., Heil, D. & Wang, G. Treatment of brackish produced water using carbon aerogel-based capacitive deionization technology. *Water Res.* **42**, 2605-2617 (2008).

7 Li, H., Liang, S., Li, J. & He, L. The capacitive deionization behaviour of a carbon nanotube and reduced graphene oxide composite. *J. Mater. Chem. A* **1**, 6335-6341 (2013).

8 Li, H. B., Pan, L. K., Nie, C. Y., Liu, Y. & Sun, Z. Reduced graphene oxide and activated carbon composites for capacitive deionization. *J Mater. Chem.* **22**, 15556-15561, (2012).

9 Wang, S. *et al.* Equilibrium and kinetic studies on the removal of NaCl from aqueous solutions by electrosorption on carbon nanotube electrodes. *Sep. Purif. Technol.* **58**, 12-16 (2007).

10 Yin, H. *et al.* Three‐Dimensional Graphene/Metal Oxide Nanoparticle Hybrids for High‐Performance Capacitive Deionization of Saline Water. *Adv. Mater.* **25**, 6270-6276 (2013).

1. * Corresponding author. Tel: 86 21 62234132; Fax: 86 21 62234321; E-mail: lkpan@phy.ecnu.edu.cn [↑](#footnote-ref-2)
